# Identification of QTL Related to ROS Formation under Hypoxia and Their Association with Waterlogging and Salt Tolerance in Barley

**DOI:** 10.3390/ijms20030699

**Published:** 2019-02-06

**Authors:** Muhammad Bilal Gill, Fanrong Zeng, Lana Shabala, Guoping Zhang, Min Yu, Vadim Demidchik, Sergey Shabala, Meixue Zhou

**Affiliations:** 1 International Centre for Environmental Membrane Biology, Foshan University, Foshan 528000, China; muhammad.bilal@utas.edu.au (M.B.G.); yumin@fosu.edu.cn (M.Y.); Dzemidchyk@bsu.by (V.D.); 2 Department of Agronomy, College of Agriculture and Biotechnology, Zhejiang University, Hangzhou 310058, China; zengfr@zju.edu.cn (F.Z.); zhanggp@zju.edu.cn (G.Z.); 3 Tasmanian Institute of Agriculture, College of Science and Engineering, University of Tasmania, Hobart, Tas 7005, Australia; L.Shabala@utas.edu.au; 4 Department of Plant Cell Biology and Bioengineering, Biological Faculty, Belarusian State University, 222030 Minsk, Belarus

**Keywords:** barley, chromosome 2H, hypoxia, QTL mapping, ROS, waterlogging tolerance

## Abstract

Waterlogging is a serious environmental problem that limits agricultural production in low-lying rainfed areas around the world. The major constraint that plants face in a waterlogging situation is the reduced oxygen availability. Accordingly, all previous efforts of plant breeders focused on traits providing adequate supply of oxygen to roots under waterlogging conditions, such as enhanced aerenchyma formation or reduced radial oxygen loss. However, reduced oxygen concentration in waterlogged soils also leads to oxygen deficiency in plant tissues, resulting in an excessive accumulation of reactive oxygen species (ROS) in plants. To the best of our knowledge, this trait has never been targeted in breeding programs and thus represents an untapped resource for improving plant performance in waterlogged soils. To identify the quantitative trait loci (QTL) for ROS tolerance in barley, 187 double haploid (DH) lines from a cross between TX9425 and Naso Nijo were screened for superoxide anion (O_2_^•^^−^) and hydrogen peroxide (H_2_O_2_)—two major ROS species accumulated under hypoxia stress. We show that quantifying ROS content after 48 h hypoxia could be a fast and reliable approach for the selection of waterlogging tolerant barley genotypes. The same QTL on chromosome 2H was identified for both O_2_^•^^−^ (*QSO.TxNn.2H*) and H_2_O_2_ (*QHP.TxNn.2H*) contents. This QTL was located at the same position as the QTL for the overall waterlogging and salt tolerance reported in previous studies, explaining 23% and 24% of the phenotypic variation for O_2_^•^^−^ and H_2_O_2_ contents, respectively. The analysis showed a causal association between ROS production and both waterlogging and salt stress tolerance. Waterlogging and salinity are two major abiotic factors affecting crop production around the globe and frequently occur together. The markers associated with this QTL could potentially be used in future breeding programs to improve waterlogging and salinity tolerance.

## 1. Introduction

Waterlogging is a worldwide constraint that considerably affects growth, development, and the distribution of plant species. In waterlogging (hypoxia, anoxia) conditions, the main factor constricting plant growth is a limited supply of oxygen to the submerged tissues; particularly in roots [1,2]. Waterlogging stress dramatically reduces available oxygen concentration to below critical levels in roots due to low diffusion rate of gases in soil and respiration of microorganisms [3,4]. Soil waterlogging gradually leads to hypoxia and with time may even result in a complete absence of oxygen (anoxia), also prompting accumulation of carbon dioxide in the root zone [5]. Under these hypoxic and anoxic conditions, oxygen deficiency limits the ability of plant roots to supply water and nutrients to shoots [6,7] and leads to disrupted plant metabolism, reduced growth rates, and lower plant yield. Salinity is the other important limiting factor in crop production and often occurs concurrently with oxygen deficiency. More than 20% of irrigated land is affected by soil salinity; this amounts to over 800 million hectares across the globe. To address the challenge of feeding more than 9.6 billion people by 2050, food production should increase by 70% [8,9]. This implies a need to improve the ability of plants to better cope with diverse abiotic factors including salinity and waterlogging.

Under waterlogged conditions, plants experience multifaceted environmental perturbations including restricted availability of oxygen and carbon dioxide, excessive accumulation of ethylene [10], and toxic elements in soil [11,12,13]. As a result, cells and tissues may be exposed to oxidative stress. Plant responses to oxygen-deprived conditions include increased generation of reactive oxygen species (ROS), essentially as superoxide radicals (O_2_^•^^−^), hydroxyl radicals (OH^•^), hydroperoxyl radicals (HO_2_^•^), and hydrogen peroxide (H_2_O_2_) [14,15]. These ROS can oxidize and trigger breakdown biological molecules, such as lipids, proteins, carbohydrates, and nucleic acids, as well as enzymatic activity [16,17]. Under oxygen-limited conditions, ROS can initially be produced by multiple mechanisms in plant roots such as plasma membrane (PM) NADPH, mitochondrial dysfunction, and after the accumulation of excess amounts of metal ions [18,19,20]. Increased iron and copper activity in the ionic and catalytically-active chelated forms (along with other transition metals) under O_2_ deprivation is widely considered as a major reason for the ROS burst via the conversion of H_2_O_2_ to extremely dangerous HO^•^ [20]. 

ROS produced under oxygen-deprived conditions also play significant roles as signalling molecules in plants in a broad range of developmental and adaptive responses to waterlogging stress. Considerable data accumulated over the years suggest that ROS production, by either PM NADPH oxidase and/or mitochondria, controls the plant adaptive responses under oxygen-limited conditions [14,21,22]. However, imbalanced production of ROS can damage cellular components and cause their dysfunction. Plants use several enzymatic and non-enzymatic sources to counter overproduced ROS. These sources include superoxide dismutase (SOD), peroxidase (POD), catalase (CAT), and ascorbate peroxidases (APX). Thus, due to the above mentioned damaging role of ROS overproduction in living tissues, the ability of the plant to produce antioxidant enzymes is generally correlated with susceptibility to environmental stresses, including waterlogging [23,24,25].

Many QTL associated with various environmental stresses have been reported in previous studies [26,27,28,29,30,31], including barley. Several QTL have been identified for waterlogging tolerance in this species based on different physiological and agronomic traits including germination rate [32,33], total root dry weight [34], chlorophyll damage index [35], grain yield [36], leaf chlorosis [27,37], survival rate [38], plant biomass indices [37,39], and photosynthetic characteristics [40]. However, each of these indices may be affected by various environmental constraints and are therefore not necessarily causally related to waterlogging stress, thus limiting their practical use. In recent studies, traits more directly related to waterlogging tolerance have been selected to identify QTL including root porosity [41], adventitious root development [42], and aerenchyma formation [41,43]. However, to the best of our knowledge, no QTL for traits associated with tissue-specific ROS productions under hypoxic conditions have been reported for any plant species, despite the essential role of oxidative damage as a major constraint imposed by waterlogging stress. 

In this study, 187 barley double haploid (DH) lines from a cross between TX9425 and Naso Nijo were screened for ROS production under hypoxia (waterlogging) stress. For the first time, we report a major QTL for both O_2_^•^^−^ and H_2_O_2_. Waterlogging stress is often accompanied by salinity and both stresses share some common mechanisms such as membrane potential maintenance and ROS detoxification. Analyses were conducted to identify the potential linkage between this trait and waterlogging and salinity tolerances. This finding may open new avenues for future breeding programs to develop more stress tolerant varieties.

## 2. Results

### 2.1. ROS (O_2_^•−^, H_2_O_2_) Production in Barley Cultivars under Hypoxia Stress

**Figure 1:**
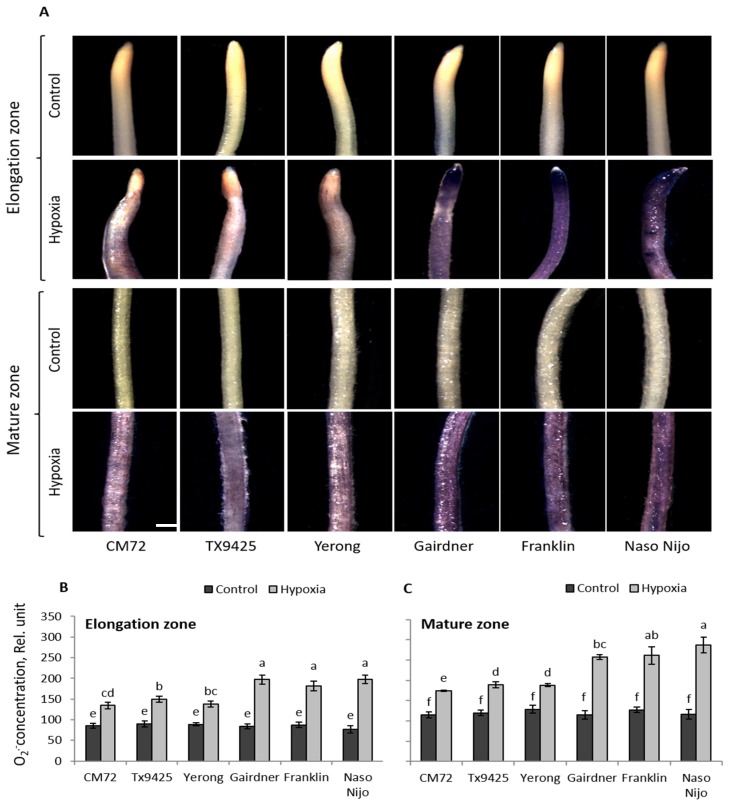
(**A**) Histochemical detection of superoxide (O_2_^•^^−^) in the elongation and mature zone in the roots of six barley cultivars differing in waterlogging tolerance. (**B**) Relative quantification of the O_2_^•^^−^ concentration in the elongation and (**C**) the mature root of barley. Image J software (NIH, Bethesda, MD, USA) was used to calculate relative (O_2_^•^^−^) concentration by targeting the fluorescence integrated density. Data are the mean ± SE; *n* = 150–250; 20–30 cells analysed for at least 6–8 individual seedlings (biological replicates). The scale bar = 1 mm. Different lowercase letters indicate the significant difference at *p* ≤ 0.05 according to Duncan’s multiple range tests.

**Figure 2:**
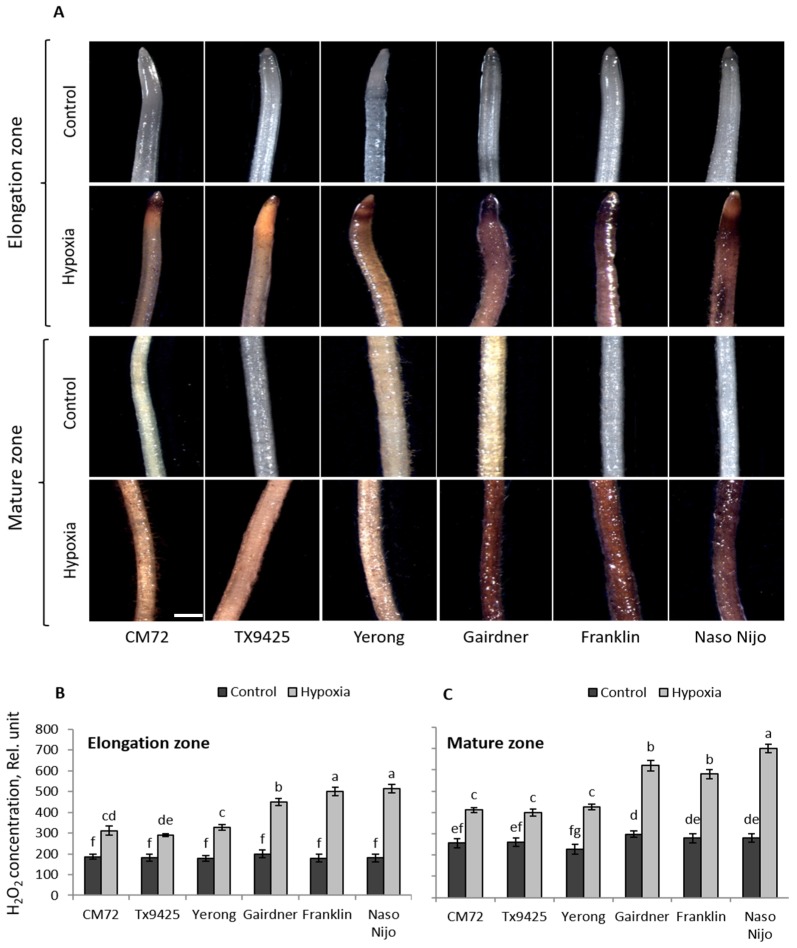
(**A**) Histochemical detection of hydrogen peroxide (H_2_O_2_) in the elongation and mature zone in the roots of six barley cultivars differing in waterlogging tolerance. (**B**) Relative quantification of the (H_2_O_2_) concentration in the elongation and (**C**) the mature root of barley. Image J software was used to calculate relative H_2_O_2_ concentration by targeting the fluorescence integrated density. Data are the mean ± SE; *n* = 150–250; 20–30 cells analysed for at least 6–8 individual seedlings (biological replicates). The scale bar = 1 mm. Different lowercase letters indicate the significant difference at *p* ≤ 0.05 according to Duncan’s multiple range tests.

Under oxygen-deprived conditions, ROS are produced in plant tissues [44,45]. To assess the suitability of the staining methodology to quantify this ROS production, six barley cultivars differing in waterlogging tolerance were used in preliminary experiments. Both O_2_^•−^ and H_2_O_2_ showed a genotypic-specific accumulation after 48 h of hypoxia stress (Figure 1 and Figure 2). 

The 48 h of hypoxia stress affected the accumulation of O_2_^•−^ radical in all cultivars, but to different extents. A higher accumulation of O_2_^•−^ in both elongation and the mature zones was observed in waterlogging sensitive cultivars Gairdner, Franklin, and Naso Nijo (Figure 1A). These visual observations were then quantified by Image J software, revealing statistically significant (*p* < 0.05) differences between sensitive and tolerant cultivars (Figure 1B,C). The production of O_2_^•−^ in both elongation and mature zones was almost 1.5- to 2-fold higher in waterlogging sensitive cultivars than in tolerant cultivars. For H_2_O_2_, the intensity of the brown color was greater in sensitive cultivars after hypoxia, suggesting more H_2_O_2_ production compared with appropriate controls (Figure 2A). Similarly, sensitive cultivars showed 2- to 2.5-fold higher accumulation of H_2_O_2_ compared with tolerant cultivars in both elongation and mature zones (Figure 2B,C) when analysed with Image J software.

### 2.2. ROS Production in DH Lines and Identification of QTL for ROS Tolerance

**Figure 3:**
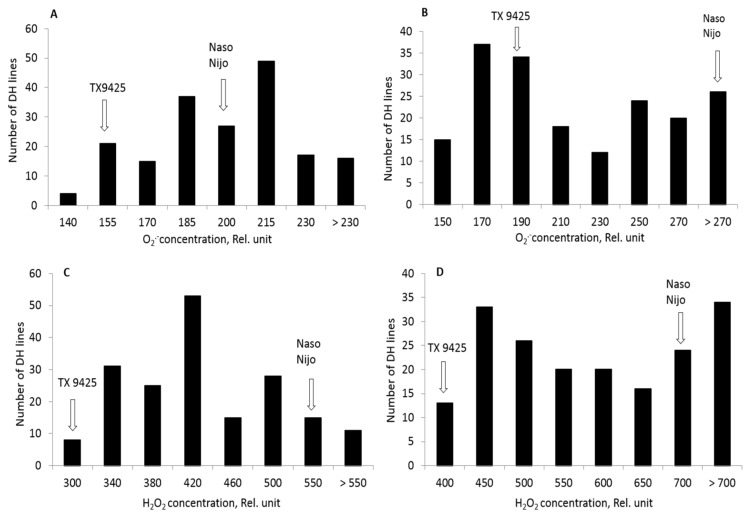
(**A**,**B**) The frequency distribution for superoxide (O_2_^•^^−^) and (**C**,**D**) hydrogen peroxide under hypoxia (0.2% agar) stress of DH lines derived from a cross between TX9425 and Naso Nijo.

**Figure 4:**
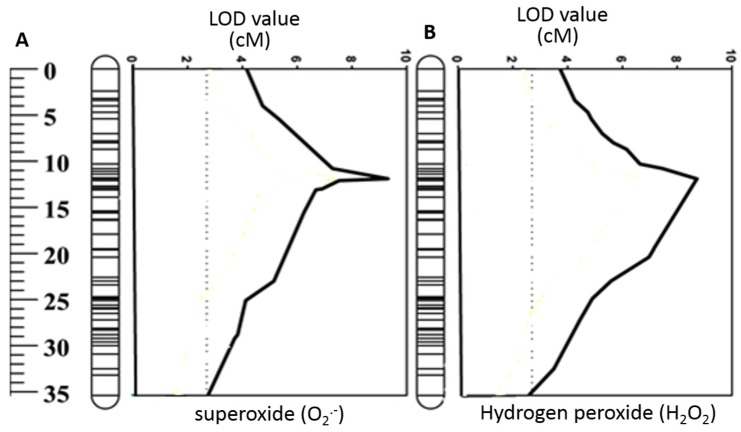
(**A**) QTL associated with superoxide (O_2_^•^^−^) radical, and (**B**) hydrogen peroxide (H_2_O_2_). For the clarity, only parts of chromosome regions are shown.

The double haploid (DH) lines derived from TX9425 and Naso Nijo were used to identify the QTL for ROS tolerance under hypoxia stress. Both parent cultivars showed a considerable difference in O_2_^•−^ and H_2_O_2_ production when measured after 48 h of hypoxia in roots (Table 1). Under hypoxia stress, the waterlogging sensitive parent Naso Nijo showed a significantly higher accumulation of O_2_^•−^ in the elongation (197) and mature (278) zones compared with the tolerant parent (149 and 189, respectively) (Table 1). Similarly, Naso Nijo showed a higher H_2_O_2_ accumulation in both elongation (515) and mature (691) zones than TX9425 (Table 1). Figure 3 shows the frequency distribution of ROS tolerance based on O_2_^•−^ and H_2_O_2_ accumulation. A continuous distribution was found for O_2_^•−^ and H_2_O_2_ accumulation in both elongation and mature zones (Figure 3). A major QTL was identified on chromosome 2H for both O_2_^•−^ in mature zone and H_2_O_2_ in elongation zone (Figure 4). The QTL were designated as (*QSO.TxNn.2H*) for O_2_^•−^ and (*QHP.TxNn.2H*) for H_2_O_2_. The closest marker was 3271162D2 for *QSO.TxNn.2H* and 3999753D2 for *QHP.TxNn.2H*, both at position 13.6 cM, explaining 23.7% and 24.1% of the phenotypic variation, respectively (Table 2). No significant QTL was identified for O_2_^•−^ in the elongation zone and H_2_O_2_ in the mature zone under hypoxia, although both showed significant difference among DH lines.

### 2.3. Contribution of ROS (O_2_^•−^, H_2_O_2_) to Waterlogging and Salinity Tolerance

**Figure 5:**
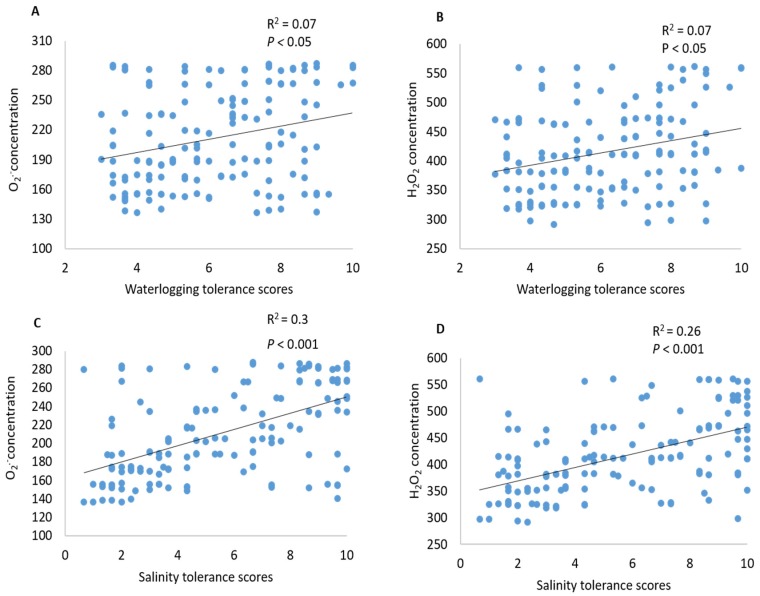
Correlations between (**A**) superoxide (O_2_^•^^−^) radical concentration in mature zone and waterlogging tolerance scores, (**B**) hydrogen peroxide (H_2_O_2_) concentration in elongation zone and waterlogging tolerance scores, (**C**) superoxide (O_2_^•^^−^) radical concentration in mature zone and salinity tolerance scores, and (**D**) hydrogen peroxide (H_2_O_2_) concentration in elongation zone and salinity tolerance scores.

**Figure 6:**
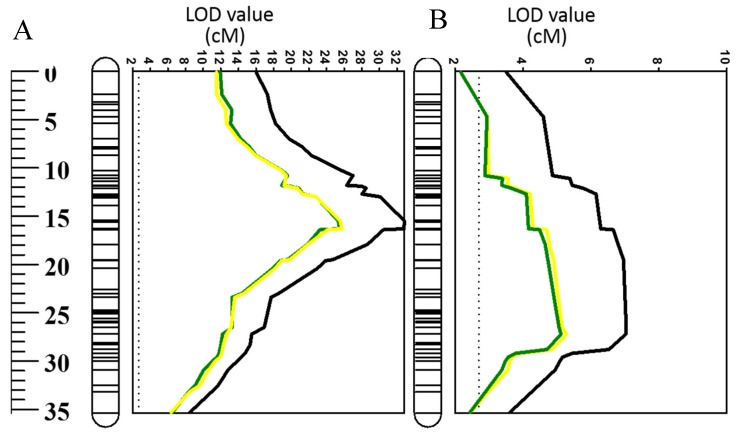
QTL associated with (**A**) salinity and (**B**) waterlogging tolerance (LOD values) on 2HS. Black line: LOD value of original QTL; green line: LOD value of QTL when superoxide (O_2_^•^^−^) in the mature zone was used as a covariate; yellow line: LOD value of QTL when hydrogen peroxide (H_2_O_2_) in elongation zone was used as a covariate.

The QTL identified for O_2_^•−^ and H_2_O_2_ in the current study were further used to examine their contribution to waterlogging and salinity tolerance by incorporating data published by Xu et al. [46]. The position of the identified QTL in the current study was the same as that for waterlogging and salinity tolerance on chromosome 2H [46]. Both O_2_^•−^ and H_2_O_2_ showed a significant (*p* ˂ 0.05) correlation with the overall waterlogging tolerance (Figure 5A,B). This was further confirmed by QTL analysis for waterlogging tolerance using O_2_^•−^ and H_2_O_2_ as covariates (Figure 6). As shown in Figure 6B, the LOD value of the QTL on 2H for waterlogging tolerance showed a slight reduction when O_2_^•−^ and H_2_O_2_ were used as covariates. The percentage of the phenotypic variation (*R*^2^) determined by the QTL reduced from 21% to 14% when O_2_^•−^ was used as a covariate, and from 21% to 14.3 when H_2_O_2_ was used as a covariate (Table 2). A close and significant correlation (*p* ˂ 0.001) with the salt tolerance was also found for both O_2_^•−^ and H_2_O_2_ (Figure 5C,D). When O_2_^•−^ and H_2_O_2_ were used as covariates, the *R*^2^ of the QTL for salt tolerance reduced from 63 to 39 when O_2_^•−^ was used as a covariate, and 63 to 41 when H_2_O_2_ was used as a covariate (Table 2).

### 2.4. Effects of Using Waterlogging and Salt Tolerance As Covariates on QTL for ROS (O_2_^•−^, H_2_O_2_)

**Figure 7:**
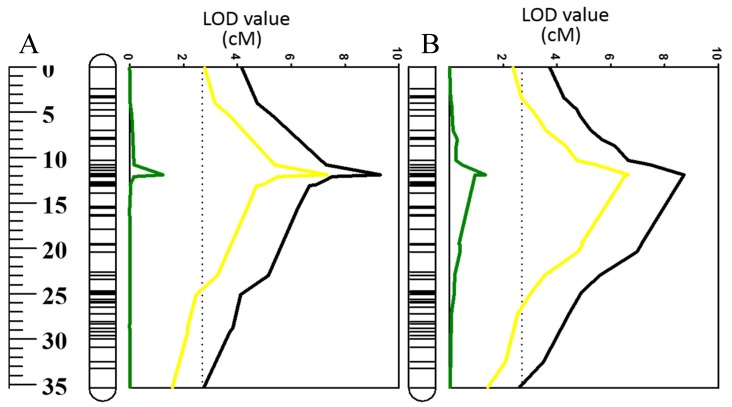
QTL associated with (**A**) superoxide (O_2_^•^^−^) in the mature zone and (**B**) hydrogen peroxide (H_2_O_2_) in elongation zone tolerance (LOD values) on 2HS. Black line: LOD value of original QTL; green line: LOD value of QTL when salinity tolerance was used as a covariate; yellow line: LOD value of QTL when waterlogging was used as a covariate.

These correlation results of O_2_^•−^ and H_2_O_2_ with waterlogging and salinity stress were further confirmed by reverse QTL analysis, i.e., analysis of QTL for O_2_^•−^ and H_2_O_2_ by using either waterlogging or salt tolerance as covariates (Figure 7; Table 2). When such analysis was conducted using waterlogging tolerance as a covariate, the significance of the QTL was reduced for O_2_^•−^ and H_2_O_2_ (Figure 7; Table 2). Similarly, the QTL for both O_2_^•−^ and H_2_O_2_ became insignificant when salt tolerance scores were used as covariates (Figure 7; Table 2).

## 3. Discussion

Waterlogging stress is one of the major abiotic factors limiting agricultural production around the globe. Hence, introducing waterlogging tolerance in field crops is crucial for sustainable food production. Waterlogging tolerance is a complex trait and can be easily affected by various environmental factors including soil properties, the extent of stress, duration of stress, and plant development stage when waterlogging occurs [47,48]. Due to these confounding factors and low efficiency of adopted direct selection methods, various indirect criteria have been used to select for waterlogging tolerance in plants. 

Many QTL have been identified for waterlogging tolerance based on different agronomic, physiological, and anatomical traits. In barley, QTL analysis for waterlogging tolerance was performed based on plant height [49], grain yield [36], plant survival [50], leaf chlorosis [27,37], and plant biomass [51] under waterlogging stress. These QTL were identified on all seven chromosomes, limiting their practical use. Also, most of these studies were based on quantitative traits, which can vary between different environments, e.g., a QTL detected in one environment could not necessarily be detected in another environment [52,53,54]. Although these traits are convenient for high throughput screening, they may not be directly related to the mechanisms conferring the tolerance. As several QTL are responsible for a trait, fine mapping of these QTL to provide reliable markers to breeders is challenging. 

Recently, a more promising approach was introduced for use when specific QTL are linked directly with the appropriate mechanisms. As most of the mechanisms are expected to be controlled by just one or two QTL enables finely mapping these mechanisms. A good example of this success is the major QTL for waterlogging tolerance on 4H in barley [37,47,55], which is due to the formation of aerenchyma under stress [43,56,57]. The gene has been fine mapped to a < 2 cM region. The closely linked molecular markers of this gene are available for breeders to use in developing waterlogging tolerance in breeding programs. In the natural environment, oxygen deficiency is not the only limitation under waterlogging stress. In future breeding programs, we need to pyramid genes related to other traits including ROS tolerance. 

Cellular ROS balance can be disturbed under stress conditions due to either enhanced production of ROS or reduced antioxidants activity in plants [15,58]. Under moderate stress conditions, ROS generation primarily acts as a regulatory and adaptive mechanism [44]. For example, ROS signaling plays an essential role in anatomical adaptations under low oxygen stress by triggering the process of aerenchyma formation [18,59]. A study showed the requirement of elevated ROS for the programmed cell death (PCD) during the development of adventitious roots in seedlings of rice [60]. However, when stress is severe, excessive generation of ROS damages cellular components and causes their dysfunction. Similarly, H_2_O_2_ contributes to activating a range of cation-permeable non-selective cation channels [61,62,63], thus affecting intracellular K^+^ and Ca^2+^ homeostasis [64], which may initiate PCD. In addition, by interacting with transition metals, H_2_O_2_ may form hydroxyl radicals that directly contribute to the activation of outward-rectifying K^+^ efflux (GORK) channels [65,66,67]. In the current experiment, hypoxia-treated roots showed a significantly higher accumulation of ROS compared with control conditions (Figure 1 and Figure 2). The accumulation of both O_2_^•^^−^ and H_2_O_2_ was higher in waterlogging sensitive cultivars than in tolerant ones (Figure 1 and Figure 2; Table 1). The DH population showed a wide range of segregation (Figure 3); the accumulation of O_2_^•^^−^ and H_2_O_2_ was correlated with both waterlogging and salinity tolerances. Major QTL were identified for both O_2_^•^^−^ (*QSO.TxNn.2H*) and H_2_O_2_ (*QHP.TxNn.2H*) (Figure 4). The QTL is located at the same position on the short arm of chromosome 2H.

Several QTL were reported at this position for different abiotic stress tolerances, which include waterlogging [46,47,68], salinity [46], and drought [69] with some being identified from the same DH population used in this study. Importantly, all these stresses are known to promote the generation and accumulation of excessive ROS in plant tissues [70,71,72]. Therefore, some common mechanisms may contribute to a close relationship between these different stress tolerances. In the current experiment, both O_2_^•^^−^ and H_2_O_2_ showed significant correlations with waterlogging and salinity tolerance (Figure 5). QTL analysis was conducted using other related traits as covariates that have been proven to be effective in confirming the relationship between different traits [69]. When O_2_^•^^−^ and H_2_O_2_ were used as covariates, the QTL for both waterlogging and salt tolerance showed a reduction in both LOD values and *R^2^* (Figure 6, Table 2). The QTL for both O_2_^•^^−^ and H_2_O_2_ became insignificant after using waterlogging or salt tolerance as covariates (Figure 7; Table 2). QTL became insignificant after using waterlogging or salt as covariates, indicating a close relationship between ROS production under stress and plants’ waterlogging/salinity tolerance. The fact that QTL were detected for several abiotic stresses at this position of chromosome 2H indicates a specific mechanism for different stress tolerances, including waterlogging and salinity tolerance.

Potassium (K^+^) is the most abundant inorganic cation in plant cells and plays a significant role in numerous physiological and metabolic processes [73,74]. K^+^ also plays a role in activating and regulating nearly 70 different metabolic enzymes in plants [75,76]. K^+^ is considered a key determinant of cell fate by acting as a trigger of the PCD under hostile conditions [77,78]. A strong correlation exists between the ability of plant tissue to retain K^+^ and waterlogging stress tolerance [79,80]. Under hypoxic conditions, K^+^ is generally leaked through GORK channels. These channels open due to membrane depolarization and ROS accumulation [64,79]. In our previous study, a major QTL (*QMP.TxNn.2H*) was identified for membrane potential with a 22% phenotypic variation [68]. The position of the QTL was the same as for the QTL in this experiment on 2H. The consistent identification of the same region on chromosome 2H in both experiments points to the presence of a specific common tolerance responsive gene.

To the best of our knowledge, this study represents the first report on the QTL mapping of waterlogging tolerance based on ROS accumulation. A major QTL was identified on chromosome 2H for both O_2_^•^^−^ and H_2_O_2_ accumulation under waterlogging stress. The position of QTL for ROS was the same as that for waterlogging and salinity tolerance. The one single QTL being identified facilitates the fine mapping of the gene responsible for waterlogging and salinity tolerance using this trait as a physiological marker. The molecular markers associated with this QTL may provide valuable evidence for marker-assisted selection (MAS) and to further breeding programs for waterlogging tolerance.

## 4. Materials and Methods

### 4.1. Plant Material

Six barley (*Hordeum vulgare* L.) cultivars contrasting in waterlogging tolerance were used in the initial part of this study. Among these cultivars, CM72, TX9425, and Yerong are tolerant; Gairdner Franklin and Naso Nijo are sensitive to waterlogging [47,81]. Seeds were acquired either from China or the Australian Winter Cereal Collection Centre (Horsham, Australia) and reproduced in the field using Tasmanian Institute of Agriculture (TIA) facilities in Launceston (Australia). For QTL analysis, data were collected from 187 DH lines originated from a cross between TX9425 and Naso Nijo. As mentioned earlier, TX9425 is a Chinese, two-rowed barley variety that is tolerant to waterlogging and salinity [81,82] and shows a few exceptional agronomic characteristics. Naso Nijo is a Japanese malting barley variety with good agronomic characteristics but is sensitive to waterlogging and salinity [55,81]. 

Seeds of DH population were grown for 3 days in 9 × 12 × 6 cm (length × width × height) containers with basic salt media (BSM) solution (0.5 mM KCl + 0.1 mM CaCl_2_, pH 5.6) at room temperature (25 ± 1 °C). Before planting, seeds were surface sterilized with 10% commercial bleach (NaClO 42 g·L^−1^; Pental Products, Shepparton, Australia) and then thoroughly washed with tap water for about 30 min. Two treatments were used in this experiment: (1) control (BSM, aerated) and (2) hypoxia (BSM solution made with 0.2% agar and bubbled with N_2_ gas). To prepare stagnant solution for hypoxia treatment, agar (Cat. No. LP0011, Oxoid, Hampshire, UK) at a ratio of 0.2% (*w*/*v*) was added to BSM and boiled until became transparent. The solution was then cooled down overnight at room temperature with an operational magnetic stirring to avoid lump formation. The agar solution for hypoxia treatment was pre-bubbled with high purity N_2_ (Cat. No. 032G, BOC Gases, Hobart, Australia) for at least 1 h before being used in the measurements.

### 4.2. Evaluation of the DH Lines for Waterlogging and Salinity Tolerance

The protocols describing the procedure and evaluation criteria for waterlogging and salt tolerance quantification were provided in previous publications from our laboratory [46]. In brief, a combined visual scoring system was used, with scoring index 0 representing no damage and index 10 specified for fully dead plants. The plants with scores 0–5 displayed the various range of chlorosis and those with scores 6 or above had a significant proportion of necrotic leaves.

### 4.3. Determination of Hydrogen Peroxide and Superoxide Radical for QTL

Prior to measurement, 3-day old seedlings of barley DH lines were treated with hypoxia solution (0.2% agar) in a container. The container was filled with hypoxia solution with coleoptile above the surface of the solution. Roots were kept under stagnant conditions for 48 h. The seedlings were then removed from hypoxia solution and ROS species accumulation was analyzed by following the given staining procedure. Hydrogen peroxide (H_2_O_2_) accumulation in barley roots of DH lines was detected after the staining with 3,3′-diaminobenzidine (DAB) according to Xu et al. [83] and Lehotai et al. [84]. In brief, fresh root apices (~0.5 cm) were incubated in 1 mg/mL DAB-HCl solution for 5 h and washed once with 2-*N*-morpholino-ethanesulfonic acid/potassium chloride (Mes/KCl) buffer (3−10 M, pH 6.15). The accumulation of superoxide anion (O_2_^•^^−^) was achieved using the nitro blue tetrazolium (NBT) staining procedure [84]. In this method, root segments (~0.5 cm) were dyed for 2 h with 0.1 mg/mL NBT in 0.2 M phosphate buffer, pH 7.6, in the dark and then washed once with a phosphate buffer. After staining, the roots were washed with distilled water for 3 to 5 times. All stained roots were observed using a Leica Fluorescence Stereomicroscope (Model MZ16 FA, Leica Microsystems, Heerbrugg, Switzerland) under visible light and photographed with a charge-coupled device (CCD) imaging system attached to the microscope. Then, images were analysed with Image J software (NIH, Bethesda, MD, USA) based on the integrated density. The background intensity of the signal was measured from an empty region with a similar size and subtracted from the whole-cell intensity to obtain relative total cell fluorescence values [85]. For each DH line and ROS species, roots segments of at least 6–8 individual seedlings were used for staining after 48 h of treatment; for each of them, between 20 and 30 cell’s (technical replicates) intensity values were averaged. For reporting purposes, relative total cell O_2_^•^^−^ and H_2_O_2_ concentration data shown in Figure 1 and Figure 2 were divided by 1000.

### 4.4. Genetic Map Construction and QTL Analysis

Leaf tissues of four-week-old seedlings of the DH population were used to extract genomic DNA. A total of 28047 DArT and 8928 SNP markers were used for genotyping. We selected 4788 markers for map construction after removing the markers with larger distortion and missing information. A new genetic map of the DH population was created using the JoinMap 4.0 software package [86]. Another software package, MapQTL 6.0, was used to perform QTL analysis [87]. In the first stage, a major QTL was detected by interval mapping (IM). The nearest marker to the major QTL was selected as a cofactor in the multiple QTL model (MQM). The logarithm of the odds (LOD) threshold values were applied to affirm the occurrence of a QTL were assessed by conducting the genome-wide permutation tests implemented in MapQTL version 6.0 using at least 1000 permutations of the original data set for each trait, resulting in a 95% LOD threshold around 3.0. QTL detected for both waterlogging and salinity tolerance were re-analysed by using different physiological traits as covariates to evaluate the effects of physiological traits on waterlogging and salinity tolerance. Finally, MAPCHART software was used to generate maps which are showing the QTL position and LOD values [88].

### 4.5. Statistical Analysis

Significant differences between means were assessed using the Duncan’s multiple range test by using the IBM SPSS Statistics 21 statistical package (IBM, New York, NY, USA). All data in the tables and figures are shown as means ± SE. Significant differences between different cultivars and/or treatments at *p* < 0.05 are represented by different lower-case letters.

## Figures and Tables

**Table 1:** Reactive oxygen species (ROS) production in the elongation and mature zones of parents and DH lines under hypoxia (0.2% agar) stress. ROS concentrations was measured in relative units (see Section 4). Data are mean values ± SE. Data labelled with different low-case letters is significant at *p* < 0.05.

Cultivar	O_2_^•^^−^ Elongation Zone	O_2_^•^^−^ Mature Zone	H_2_O_2_ Elongation Zone	H_2_O_2_ Mature Zone
TX9425	149 ± 7b	189 ± 7b	290 ± 14c	400 ± 17b
Naso Nijo	197 ± 10a	278 ± 16a	515 ± 19a	691 ± 18a
DH lines	194 ± 14a	212 ± 28b	416 ± 42b	576 ± 73a
DH lines range	137–232	135–287	287–561	358–777

**Table 2:** QTL on 2HS for superoxide radical (O_2_^•^^−^), hydrogen peroxide (H_2_O_2_), salt, and waterlogging tolerance detected in a DH population of TX9425 × Naso Nijo. LOD = logarithm of the odds.

Trait	Linkage Group	Nearest Marker	Position (cM)	LOD	R^2^ (%)	Co-Variate
O_2_^•^^−^ mature zone	2H	3271162D2	13.6	8.7	23.7	
		No QTL identified				waterlogging
		No QTL identified				salt
H_2_O_2_ elongation zone	2H	3999753D2	13.6	8.9	24.1	
		No QTL identified				waterlogging
		No QTL identified				salt
Waterlogging tolerance	2H	3264866S2	9.2	7.6	21	
	2H	3264866S2	9.2	5.6	14.8	O_2_
	2H	3264866S2	9.2	5.4	14.3	H_2_O_2_
Salt tolerance	2H	3257177S2	7.8	32.7	63.7	
	2H	3257177S2	7.8	26.7	39.4	O_2_
	2H	3257177S2	7.8	26.6	41.3	H_2_O_2_
